# Indicators for the prescription of foot and ankle orthoses for children with Charcot-Marie-Tooth disease

**DOI:** 10.1186/1757-1146-5-S1-P23

**Published:** 2012-04-10

**Authors:** Grant Scheffers, Claire Hiller, Kathryn Refshauge, Joshua Burns

**Affiliations:** 1 Faculty of Health Sciences, The University of Sydney, NSW, Australia; 2 Institute for Neuroscience and Muscle Research, Children’s Hospital at Westmead, Sydney, Australia

## Background

Charcot-Marie-Tooth disease (CMT) is the most common inherited peripheral neuropathy and is associated with foot deformity, gait abnormalities and functional impairment. Orthoses are often prescribed for children with CMT, yet the indication and type of prescription is usually based on clinical judgement due to the lack of high quality research in this field. Therefore, the aims of this paper were to review the indications of commonly prescribed foot and ankle orthoses, and formulate a clinical algorithm for the optimal prescription of foot and ankle orthoses for children with CMT.

## Materials and methods

We searched MEDLINE (from January 1966), EMBASE (from January 1980), CINAHL (from January 1982), AMED (from January 1985), Cochrane Neuromuscular Disease Group Specialized Register, and reference lists of articles.

## Results

Table 1 shows a clinical algorithm for prescribing foot and ankle orthoses for children with CMT. In general, in-shoe orthoses are indicated for affected children with pes cavus deformity, foot pain and/or mild balance impairments. Ankle-foot orthoses are indicated for children with pes cavus, foot drop, foot and ankle muscle weakness and/or ankle equinus, and moderate-severe balance impairments and/or difficulty walking (self-reported clumsy gait, frequent trips/falls) and gait abnormalities (slower speed, shorter step length, wider base of support).

**Table 1:** Clinical algorithm for prescribing foot and ankle orthoses for children with CMT

Impairments and activity limitations	Orthoses
Pes cavus and foot pain	Foot orthoses
Pes cavus and poor balance	UCBL* orthoses
Pes cavus and poorer balance (not corrected by UCBL* orthoses)	Supramalleolar orthoses
Pes cavus and poorer balance (not corrected by supramalleolar AFOs†)	Hinged AFOs†
Foot drop and poor walking	Posterior leaf spring AFOs†
Foot drop, poor walking, pes cavus, and poor balance	Hinged AFOs† with PF‡ stops
Global weakness of foot/ankle muscles and poor walking and/or balance (with/without pes cavus and/or foot drop)	Hemispiral AFOs†
Global weakness of foot/ankle muscles and poorer walking and/or balance (not corrected by hemispiral AFOs†, with/without pes cavus and/or foot drop)	Solid AFOs†
Pes cavus and/or ankle equinus (≥ 0°, not corrected by hinged AFOs† with/without PF‡ stops)	Solid AFOs†

* University of California Biomechanics Laboratory; † ankle-foot orthoses; ‡ plantarflexion

## Conclusions

A clinical algorithm is proposed to guide the prescription of orthoses for children with CMT. Further research is required to determine the efficacy of different foot and ankle orthoses, and the predictive ability of the proposed clinical algorithm to improve foot deformity, gait abnormalities and disability in childhood CMT.

